# Evolutionary conservation of acylplastoquinone species from cyanobacteria to eukaryotic photosynthetic organisms of green and red lineages

**DOI:** 10.3389/fpls.2025.1569038

**Published:** 2025-03-24

**Authors:** Ryo Ito, Mizuki Endo, Motohide Aoki, Shoko Fujiwara, Norihiro Sato

**Affiliations:** School of Life Sciences, Tokyo University of Pharmacy and Life Sciences, Hachioji, Tokyo, Japan

**Keywords:** acylplastoquinol, primary endosymbiotic algae, secondary endosymbiotic algae, cyanobacteria, LC-MS/MS^2^ analysis, plastoquinone-B, seed plants, *slr2103*

## Abstract

Plastoquinone plays a crucial role in the photosynthetic electron transport system as an electron carrier, transferring electrons from photosystem II to cytochrome b_6_f complexes. Certain cyanobacteria acylate plastoquinone derivatives, plastoquinol, the reduced form of plastoquinone, and/or plastoquinone-C, the hydroxylated form of plastoquinone to synthesize newly found cyanobacterial lipids, acylplastoquinol and acylplastoquinone-C, the latter of which is known as plastoquinone-B in seed plants. The cyanobacterial genes, *slr2103* in *Synechocystis* sp. PCC 6803 and its ortholog in *Synechococcus* sp. PCC 7002, encode a bifunctional acyltransferase for the synthesis of both acylplastoquinol and plastoquinone-B. Despite conservation of *slr2103* orthologs across a wide range of cyanobacteria, only four cyanobacterial strains, including the two mentioned above, have been identified as producing acylplastoquinol and/or plastoquinone-B. Moreover, the extent to which acylplastoquinone species are distributed in eukaryotic photosynthetic organisms that lack *slr2103* orthologs remains largely unknown. Using LC-MS/MS^2^ analysis of total cellular lipids, this study demonstrates that acylplastoquinol and plastoquinone-B are conserved not only in cyanobacteria with *slr2103* orthologs but also in eukaryotic photosynthetic organisms lacking these orthologs, including primary and secondary endosymbiotic algae, and a seed plant. Notably, in eukaryotic photosynthetic organisms as well as in cyanobacteria, these acylplastoquinone species are predominantly esterified with saturated fatty acids. The evolutionary conservation of these acylplastoquinone species suggests replacement of *slr2103* orthologs by alternative gene(s) responsible for their synthesis at least once after the primary endosymbiotic event in the evolution of photosynthetic organisms. The persistent conservation of acylplastoquinone species throughout the evolution likely reflects their critical physiological roles.

## Introduction

Cyanobacteria include strains that are amenable to metabolic engineering, such as *Synechocystis* sp. PCC 6803 (herein referred to as *Synechocystis*), which have been shown to be promising producers of photosynthesis-based high-value-added products (Agarwal et al., 2022). These products include free fatty acids, which are utilized as raw materials for biodiesel fuel production. Unlike in cyanobacteria, biofuel production in algae focuses on triacylglycerol (TG), a molecule esterified with three fatty acid molecules, in view of algal ability to accumulate TG (Li-Beisson et al., 2019): some oleaginous microalgae conditionally hyperaccumulate TG to account for up to half of their dry cell weight (Bondioli et al., 2012; Hayashi et al., 2017). Interestingly, a lipid exhibiting the same Rf value as TG in TLC analysis was detected in *Synechocystis* [TG-like lipid; (Aizouq et al., 2020; Tanaka et al., 2020)], although it was present at a minor level [approximately 1% of total lipids based on fatty acid content; (Kondo et al., 2023b)]. Eventually, three independent research groups identified acylation products of plastoquinone (PQ) derivatives (acylplastoquinone species) in cyanobacteria, including that corresponded to this TG-like lipid (Ishikawa et al., 2023; Kondo et al., 2023b; Kondo et al., 2023a; Mori-Moriyama et al., 2023).

Plastoquinone (PQ) is a ubiquitous electron carrier in oxygenic photosynthetic organisms, participating in the photosynthetic electron transport chain. PQ is reduced to plastoquinol (PQH_2_) in the photosystem II (PSII) complex and oxidized back to PQ by the cytochrome b₆f complex (Havaux, 2020). One acylplastoquinone species, corresponding to the TG-like lipid, was identified as acylplastoquinol (APQ), i.e., PQH_2_ acylated with 16:0 or 18:0 fatty acids at one of its two hydroxyl groups. APQ has thus far been reported in two unicellular cyanobacteria, *Synechocystis* and *Synechococcus* sp. PCC 7002 (*Synechococcus* 7002), and two filamentous ones, *Nostoc punctiforme* PCC 73102 (*Nostoc*) and *Anabaena* sp. PCC 7120 (*Anabaena*) (Ishikawa et al., 2023; Kondo et al., 2023b; Kondo et al., 2023a; Mori-Moriyama et al., 2023). Apart from its role in redox reactions during photosynthesis, PQ is hydroxylated at a site on its isoprenyl chain to form hydroxylated PQ (PQC), which is generated through the action of singlet oxygen (^1^O_2_), a reactive oxygen species produced as a byproduct of photosynthesis (Kruk and Szymańska, 2021). In *Synechocystis* and *Synechococcus* 7002, another acylplastoquinone species was identified as PQC acylated with 16:0 or 18:0 fatty acids (Kondo et al., 2023b; Kondo et al., 2023a), which has long been known as PQB in seed plants (Das et al., 1967; Crane, 2010).

Along with the findings of APQ and PQB, the genes responsible for their metabolism have been also identified in cyanobacteria. Homologs of the type-2 diacylglycerol acyltransferase gene (DGAT2), which catalyzes acylation of diacylglycerol for TG synthesis in eukaryotes, were found in over 100 cyanobacterial strains with exclusion of oceanic *Prochlorococcus and Synechococcus* strains, representing approximately one-fourths of sequenced cyanobacterial genomes (Kondo et al., 2023b). The *DGAT2*-homologs were found in above four APQ- and/or PQB-containing cyanobacterial strains (e.g., *slr2103* in *Synechocystis*), whereas the homolog was absent in *Synechococcus* sp. PCC 7942 that possesses no acylplastoquinone species (*Synechococcus* 7942) (Aizouq et al., 2020; Tanaka et al., 2020; Ishikawa et al., 2023; Kondo et al., 2023b; Mori-Moriyama et al., 2023). Disruption of *slr2103* in *Synechocystis* (Δ*slr2103*) led to the complete absence of APQ (Ishikawa et al., 2023; Kondo et al., 2023b), while, overexpression of *slr2103* in *Synechococcus* 7942 resulted in the appearance of APQ (Kondo et al., 2023b). This demonstrated that *slr2103* is indispensable for APQ synthesis in *Synechocystis*, and that this single gene would be sufficient to confer APQ synthesis capability in cyanobacteria. Gain- and loss-of-function analyses further revealed that *slr2103* is also essential for PQB synthesis (Kondo et al., 2023b). These findings, together with conservation of DGAT2 motifs in Slr2103 protein, provide *in vivo* evidence that *slr2103* encodes a bifunctional acyltransferase capable of transferring saturated fatty acids to both PQH_2_ and PQC (Kondo et al., 2023b). This conclusion is further supported by observations of APQ and PQB loss in *Synechococcus* 7002 disruptant as to its *slr2103* ortholog, SYNPCC7002_A0918 (Kondo et al., 2023a). Later identification of an APQ lipase (APL) gene in *Synechocystis*, which cleaves the fatty acid residue from APQ to regenerate PQH_2_, adds further insights into APQ metabolism (Jimbo et al., 2024).

Despite the ubiquitous presence of PQ in both prokaryotic and eukaryotic photosynthetic organisms, the taxonomic distribution of acylplastoquinone species remains poorly understood. APQ has been identified in only four cyanobacterial strains, while PQB is known to be present in just two of these strains, alongside its established prevalence in seed plants (Crane, 2010). This study investigates acylplastoquinone species in *slr2103* ortholog-containing cyanobacteria beyond those previously reported, and also in eukaryotic photosynthetic organisms lacking *slr2103* orthologs, including primary and secondary endosymbiotic algae and a seed plant.

## Materials and methods

### Biological materials and culturing of photosynthetic microbes

The photosynthetic microbes used in this study were as follows: three cyanobacteria—*Arthrospira platensis* (herein referred to as *Arthrospira*), *Oscillatoria rosea* Utermöhl (herein referred to as *Oscillatoria*), and *Synechocystis*—and a red alga, *Cyanidioschyzon merolae* (Hirabaru et al., 2010), two green algae, *Chlorella kessleri* (Oishi et al., 2022) and *Chlamydomonas reinhardtii* (Sato et al., 1995), and a haptophyte, *Pleurochrysis haptonemofera* (Takahashi et al., 2002). Cyanobacterial cells were cultured in liquid medium at 30°C in 50 mL glass tubes with air bubbling and light illumination at 50 µE·m^−2^·s^−1^ (Kondo et al., 2023b). The media used were SOT for *Arthrospira* (Holm-Hansen et al., 1954), f/2 for *Oscillatoria* (Guillard and Ryther, 1962), and BG11 for *Synechocystis* (Kondo et al., 2023b). Red and green algal cells were cultured in liquid medium with air bubbling, similar to the method used for cyanobacteria. The cultures conditions were as follows: 40°C with light intensity of 100 µE·m^−2^·s^−1^ for *C. merolae*, and 30°C with light intensity of 200 µE·m^−2^·s^−1^ for *C. kessleri* and *C. reinhardtii*. The culture media used were modified Allen’s autotrophic medium (Hirabaru et al., 2010) for *C. merolae*, 4-fold diluted Gamborg’s B5 medium (Oishi et al., 2022) for *C. kessleri*, and 3/10 HSM medium (Sato et al., 1995) for *C. reinhardtii*. *Pleurochrysis haptonemofera* cells were cultured at 20°C in a glass bottle containing ESM medium (Takahashi et al., 2002), with air bubbling and light illumination at 30 µE·m^−2^·s^−1^. Cells at the late logarithmic growth phase (OD_730_ = 0.5) were harvested by centrifugation (3,000×g, 15 min) at 4°C, and the cell pellets were stored at -80°C until use. Additionally, spinach (*Spinacia oleracea*) was purchased from a local market for use as a seed plant.

### LC-MS/MS^2^ analysis of lipids

Total lipids were extracted from frozen cells of the respective photosynthetic microbes, or from liquid-nitrogen-frozen leaves of *Spinacia oleracea*, following the method of Bligh and Dyer (1959), as previously described (Kondo et al., 2023b; Kondo et al., 2023a). The total lipids were analyzed using an LC-QqQ(LIT)-MS/MS system (Kondo et al., 2023b; Kondo et al., 2023a), consisting of a Shimadzu LC-20A Prominence series HPLC (Kyoto, Japan) and a 3200 QTRAP hybrid triple quadrupole/linear ion trap mass spectrometer with a Turbo V™ ion source (Sciex, Concord, ON, Canada). Lipid signals were detected via enhanced mass scanning (EMS) in positive electrospray ionization (ESI+) mode. Acyl-plastoquinone species were analyzed via tandem MS² analysis as part of lipid profiling workflow. Information-dependent acquisition (IDA) combined with enhanced product ion (EPI) scans in positive ion mode was employed to enable the identification and structural characterization of target lipid species. Data acquisition and processing were performed using Analyst software (version 1.7.3, Sciex). Lipid peak signals corresponding to ammonium adduct ion peaks of the target molecules were integrated based on their extracted ion chromatograms (XICs). To ensure comparability between samples, each lipid signal’s integrated XIC peak area was normalized to the total integrated lipid signal area [retention time of 2–18 min, *m*/*z* 300–1200; (Kondo et al., 2023a)], accounting for variations in sample loading. The values are expressed as the mean ± SD of three biological replicates, each with two measurements.

## Results

### The presence of APQ and PQB in cyanobacteria containing *slr2103* orthologs

We propose that Slr2103 or its orthologs in cyanobacteria function as bifunctional acyltransferases responsible for the synthesis of both APQ and PQB (Kondo et al., 2023b; Kondo et al., 2023a). Among over 100 cyanobacteria identified as possessing *slr2103* or its orthologs, only *Synechocystis* and *Synechococcus* 7002 have been demonstrated to synthesize both APQ and PQB (Kondo et al., 2023b; Kondo et al., 2023a). Considering that these two strains are unicellular, we investigated acylplastoquinone species in two filamentous cyanobacteria, *Arthrospira* and *Oscillatoria*, which also contain *slr2103* orthologs (SPLC1_S531670 and NIES208_13960, respectively), through LC-MS/MS² analysis of total cellular lipids, as previously described (Kondo et al., 2023b; Kondo et al., 2023a).

**Figure 1:**
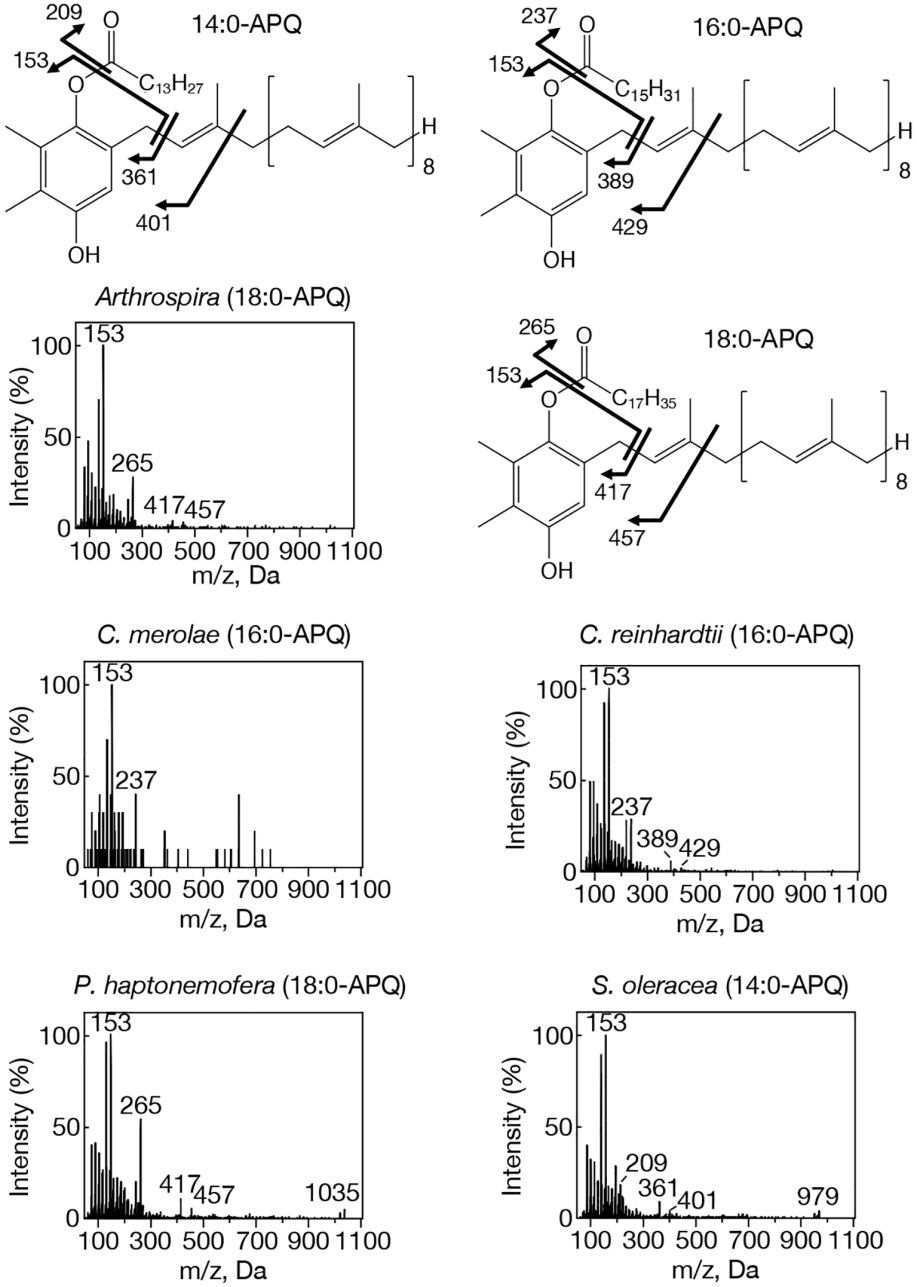
MS^2^ spectra of representative APQ species with NH_4_^+^ adducts in prokaryotic and eukaryotic oxygenic photosynthetic organisms. *Arthrospira*, 18:0-APQ; *C. merolae*, 16:0-APQ; *C. reinhardtii*, 16:0-APQ; *P. haptonemofera*, 18:0-APQ; *S. oleracea*, 14:0-APQ. NH_4_^+^-adducted 14:0-, 16:0-, and 18:0-APQ species were searched for by target LC-MS analysis as exhibiting m/z 979, m/z 1007, and m/z 1035, respectively. One of two possible chemical structures of 14:0-, 16:0- or 18:0-APQ is shown. The fragment ion, m/z 153, was detected in 14:0- and 18:0-APQ as well as in 16:0-APQ. Other three fragment ions shown in 16:0-APQ, m/z 237, m/z 389, and m/z 429, are respectively smaller by m/z 28 in 14:0-APQ whereas they are respectively greater by m/z 28 in 18:0-APQ.

**Figure 2:**
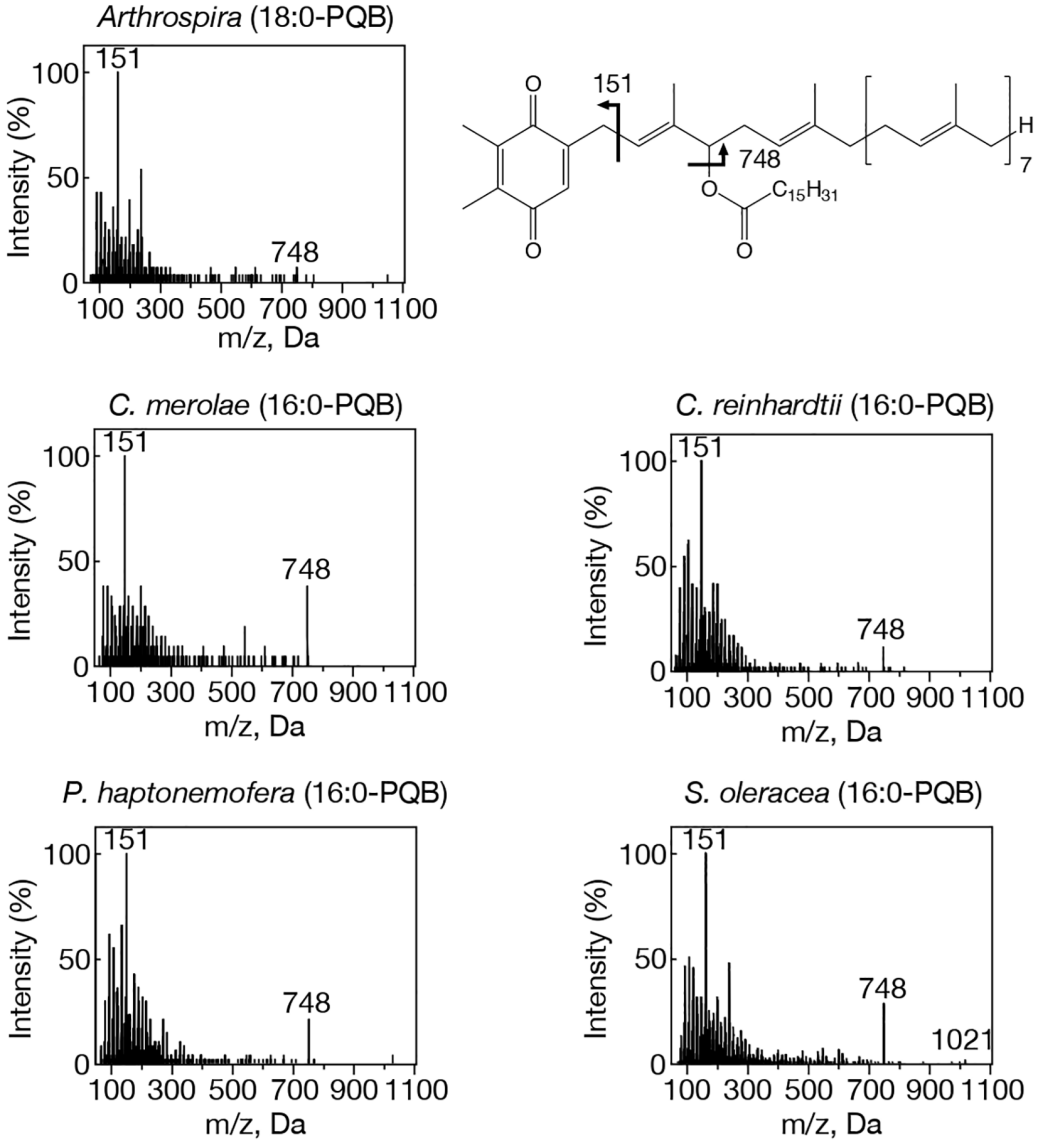
MS^2^ spectra of representative PQB species with NH_4_^+^ adducts in prokaryotic and eukaryotic oxygenic photosynthetic organisms. *Arthrospira*, 18:0-PQB; *C. merolae*, 16:0-PQB; *C. reinhardtii*, 16:0-PQB; *P. haptonemofera*, 16:0-PQB; *S. oleracea*, 16:0-PQB. NH_4_^+^-adducted 16:0- and 18:0-PQB were searched for by target LC-MS analysis as exhibiting m/z 1021 and m/z 1049, respectively. The chemical structure of PQB is shown. Two fragment ions, m/z 151 and m/z 748, were commonly detected in 16:0- and 18:0-PQB.

**Figure 3:**
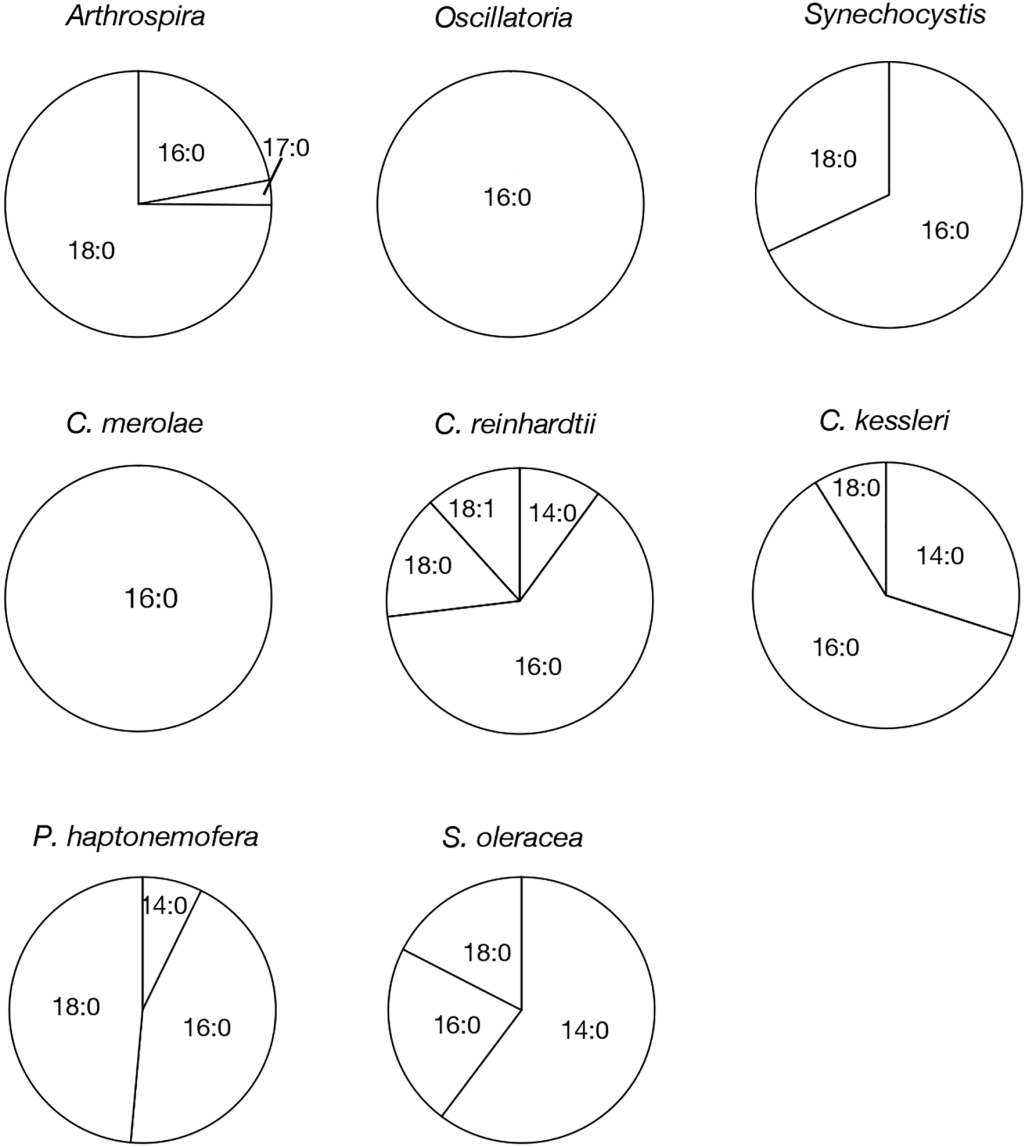
Molecular species composition of APQ in prokaryotic and eukaryotic oxygenic photosynthetic organisms. The composition of individual APQ molecular species was estimated based on their signal intensities from LC-MS spectra, relative to the total lipids (**Supplementary Figure 2**).

**Figure 4:**
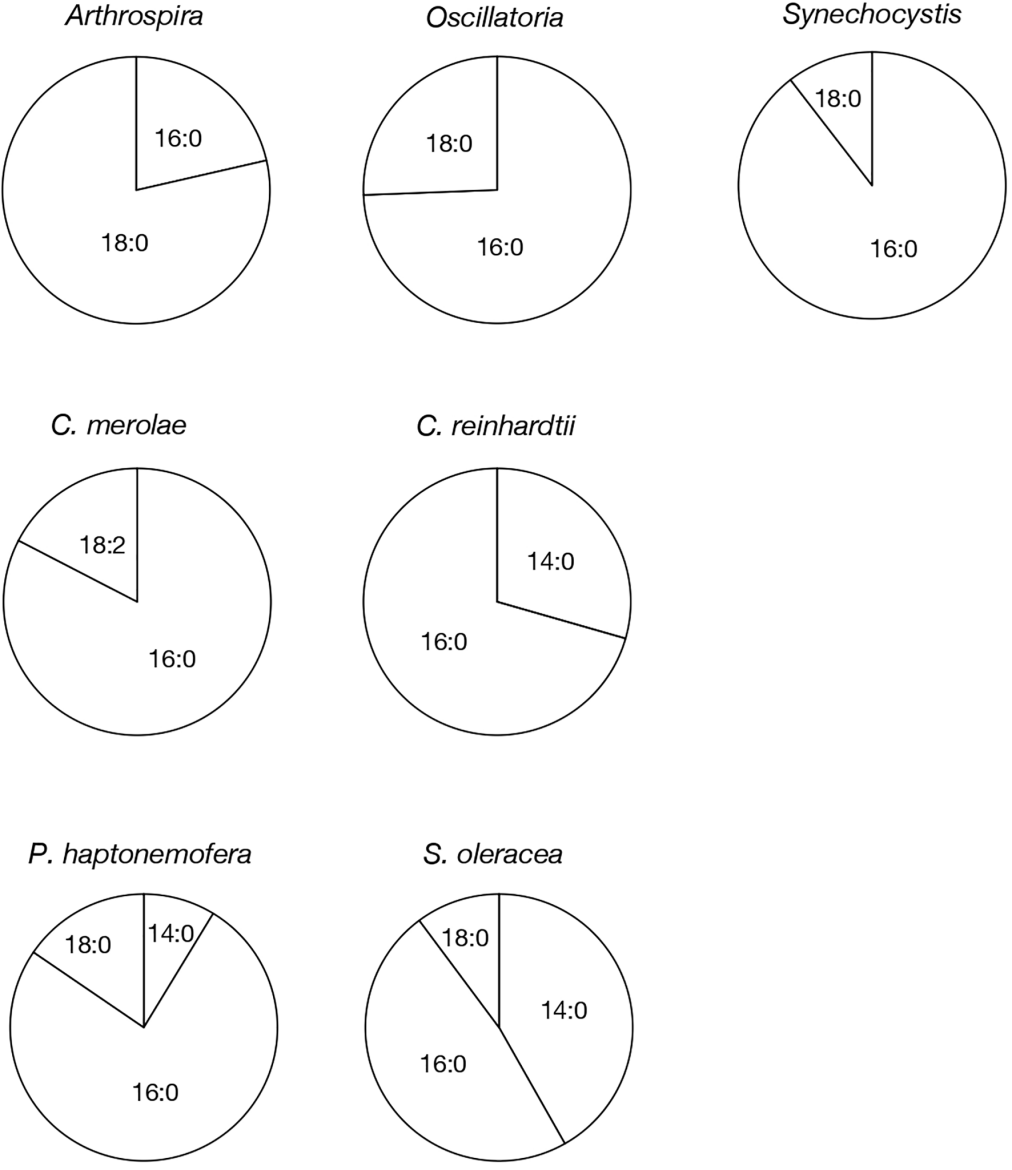
Molecular species composition of PQB in prokaryotic and eukaryotic oxygenic photosynthetic organisms. The composition of individual PQB molecular species was estimated based on their signal intensities from LC-MS spectra, relative to the total lipids (**Supplementary Figure 3**).

Based on their m/z values, retention times in LC-MS chromatograms, and MS² spectra, we identified 16:0- and 18:0-APQ (**Figure 1**; **Supplementary Figure S1**) and 16:0- and 18:0-PQB (**Figure 2**; **Supplementary Figure S1**) in *Arthrospira*. Additionally, another APQ molecular species, 17:0-APQ was identified (**Supplementary Figure S1**). Quantitative analysis of the APQ species (**Figure 3**) using signal intensities in the LC-MS spectrum (**Supplementary Figure S2**) revealed that APQ consisted primarily of 16:0- and the more abundant 18:0-species (22.1 ± 2.6% and 74.9 ± 12.9%, respectively). As a minor APQ species, 17:0-APQ amonted at 3.0 ± 0.7%. Similarly, PQB comprised 16:0- and the more abundant 18:0-species (21.4 ± 0.1% and 78.6 ± 0.3%, respectively) (**Figure 4**; **Supplementary Figure S3**).

*Oscillatoria* contained only 16:0-APQ (**Supplementary Figure S1**) as its APQ species (**Figure 3**; **Supplementary Figure S2**) and two PQB species, 16:0- and 18:0-PQB (**Supplementary Figure S1**), where 16:0-PQB was more abundant than 18:0-PQB (75.2 ± 24.5% and 24.8 ± 4.6%, respectively) (**Figure 4**; **Supplementary Figure S3**). In *Synechocystis* as well as in *Oscillatoria*, the 16:0-species quantitatively exceeded the 18:0-species in both APQ and PQB (**Figures 3**, **4**; **Supplementary Figures S2**, **S3**; 16:0-APQ: 71.6 ± 19.6%, vs. 18:0-APQ: 28.4 ± 10.0%; 16:0-PQB: 61.5 ± 4.7%, vs. 18:0-PQB: 38.5 ± 1.6%). Collectively, *Arthrospira* and *Oscillatoria* exhibited acyl-plastoquinone profiles similar to those in *Synechocystis*: both APQ and PQB were acylated with saturated fatty acids, 16:0 and/or 18:0, although the relative abundance of 16:0- and 18:0-species varied depending on the cyanobacterial strain. The *slr2103* orthologs in *Arthrospira* and *Oscillatoria* likely serve as bifunctional acyltransferases, transferring saturated fatty acids to PQH_2_ and PQC for the biosynthesis of APQ and PQB, respectively.

### Prevalent conservation of APQ and PQB in eukaryotic photosynthetic organisms that lack *slr2103* orthologs

Our study on acylplastoquinone species was then extended to eukaryotic photosynthetic organisms, all of which lack *slr2103* orthologs. We began by examining three primary endosymbiotic algae: the red alga *C. merolae* and two green algae, *C. reinhardtii* and *C. kessleri*. In *C. merolae*, we identified the following acylplastoquinone species through LC-MS/MS² analysis of total cellular lipids: 16:0-APQ (**Figure 1**) as the sole APQ species (**Figure 3**; **Supplementary Figure S2**) and 16:0- and 18:2-PQB (**Figure 2**; **Supplementary Figure S1**) as PQB species. The 16:0-PQB species was more abundant than 18:2-PQB (82.6 ± 3.9% and 17.4 ± 1.2%, respectively; **Figure 4**; **Supplementary Figure S3**).

In *C. reinhardtii*, we identified 14:0-, 16:0-, 18:0-, and 18:1-species of APQ (**Figure 1**; **Supplementary Figure S4**). The 16:0-species was the most abundant (63.1 ± 3.4%), followed by 18:0-, 18:1-, and 14:0-species (15.2 ± 0.6%, 11.7 ± 0.9%, and 10.0 ± 0.5%, respectively; **Figure 3**; **Supplementary Figure S2**). For PQB, 14:0- and 16:0-species were identified (**Figure 2**; **Supplementary Figure S4**), with 16:0-species being more abundant than 14:0-species (70.6 ± 7.6% and 29.4 ± 3.6%, respectively; **Figure 4**; **Supplementary Figure S3**).

Similarly, in *C. kessleri*, 14:0-, 16:0-, and 18:0-APQ species were identified (**Supplementary Figure S4**), with 16:0-APQ being the most prevalent (61.1 ± 2.9%), followed by 14:0-APQ (30.0 ± 1.8%) and 18:0-APQ (8.9 ± 0.6%; **Figure 3**; **Supplementary Figure S2**). Unlike *C. reinhardtii*, *C. kessleri* lacked 18:1-APQ. Concerning PQB, the ion of 16:0-species only was detected on LC-MS chromatogram, judged from its m/z value and retention time. However, 16:0-PQB in *C. kessleri* was excluded in **Figure 4**, in view of its weak signal such that the lipid ion could not be properly characterized through MS^2^ analysis. Collectively, in the red and green algae examined, the 16:0-species prevailed in both APQ and PQB.

Lastly, we investigated the secondary endosymbiotic alga *P. haptonemofera* and the seed plant *S. oleracea*, which evolutionarily originated from red and green algae, respectively. In *P. haptonemofera*, we identified 14:0-, 16:0-, and 18:0-APQ species (**Figure 1**; **Supplementary Figure S5**). The 16:0- and 18:0-species dominated APQ (44.2 ± 8.5% and 48.6 ± 9.0%, respectively), with 14:0-APQ making up the remainder (7.2 ± 0.6%; **Figure 3**; **Supplementary Figure S2**). For PQB, 14:0-, 16:0-, and 18:0-species were detected (**Figure 2**; **Supplementary Figure S5**), with the 16:0-species being the most abundant (75.8 ± 11.7%), followed by 18:0- and 14:0-PQB (15.5 ± 2.6% and 8.7 ± 0.9%, respectively; **Figure 4**; **Supplementary Figure S3**). In *S. oleracea*, we identified 14:0-, 16:0-, and 18:0-APQ species (**Figure 1**; **Supplementary Figure S5**), with 14:0-APQ being the most abundant (60.2 ± 18.0%), followed by 16:0-APQ (22.3 ± 6.1%) and 18:0-APQ (17.4 ± 5.0%; **Figure 3**; **Supplementary Figure S2**). Consistent with a previous report on *S. oleracea* (Kruk et al., 1998), we identified 14:0-, 16:0-, and 18:0-PQB species (**Figure 2**; **Supplementary Figure S5**). Among these, 14:0- and 16:0-PQB were the two major species (41.8 ± 13.9% and 48.0 ± 13.9%, respectively), followed by 18:0-PQB (10.2 ± 2.4%; **Figure 4**; **Supplementary Figure S3**). Similar to cyanobacteria and primary endosymbiotic algae, both *P. haptonemofera* and *S. oleracea* exhibited APQ and PQB species acylated predominantly with saturated fatty acids.

## Discussion

### Acyltransferase genes for APQ and PQB synthesis

This study revealed that both APQ and PQB, which are acylated mainly by saturated fatty acids such as 16:0 and 18:0, are distributed across a wide taxonomic range of oxygenic photosynthetic organisms, as follows: *slr2103-*orthologs possessing cyanobacteria, and *slr2103*-orthologs lacking eukaryotic photosynthetic organisms from primary endosymbiotic red and green algae to a secondary endosymbiotic alga and a seed plant, which are evolutionarily derived from red and green algae, respectively. Tanikawa et al. (2025) recently demonstrated that PQB is also present in two other *slr2103*-orthologs possessing cyanobacteria, *Nostoc* and *Anabaena*, where APQ had previously been identified. These findings support our hypothesis that *slr2103* orthologs encode a bifunctional acyltransferase responsible for both APQ and PQB synthesis. Additionally, Tanikawa et al. (2025) reported that two cyanobacterial strains lacking *slr2103* orthologs produced APQ but not PQB, suggesting the involvement of unknown genes in APQ-specific synthesis in these strains. The expression of *slr2103* orthologs in the respective cyanobacterial strains used in our study and theirs will be investigated in future research.

Initially, *slr2103* was identified as a homolog of the gene for DGAT2 or phytol ester synthase (PES) (Aizouq et al., 2020; Tanaka et al., 2020). Future studies should focus on identifying the acyltransferases or their corresponding genes responsible for APQ and PQB synthesis in eukaryotic photosynthetic organisms, considering DGAT2 and PES as potential candidates. In this context, it is notable that 14:0 was found in APQ and PQB in green algae, the secondary symbiotic alga, and a seed plant, while it was excluded in cyanobacteria and red algae. These differences may be attributed to variations in the acyltransferases responsible for acylplastoquinone species synthesis among photosynthetic organisms.

### APQ and PQB synthesis - acyl-acceptor synthesis in cyanobacteria and eukaryotic photosynthetic organisms

APQ and PQB are acylated derivatives of PQH_2_ and PQC, respectively (Kondo et al., 2023b; Kondo et al., 2023a). PQH_2_ is produced in the thylakoid membranes through photosynthetic electron transport in all oxygenic photosynthetic organisms (Havaux, 2020). Meanwhile, PQC is formed non-enzymatically through the action of ^1^O_2_ commonly generated at PSII. Accordingly, the presence of PQC has been widely observed in cyanobacteria, green algae, and seed plants (Kruk and Strzałka, 1998; Kruk and Trebst, 2008; Kondo et al., 2023b; Kondo et al., 2023a). In *A. thaliana* plastids, PQC was predominantly localized in the thylakoid membranes (Ksas et al., 2018), suggesting that PQC is synthesized in the thylakoid membranes where PSII operates and remains localized there. In eukaryotic photosynthetic organisms, PQH_2_ and PQC would be secured within plastids for APQ and PQB synthesis.

### APQ and PQB synthesis - acyl-donor synthesis in cyanobacteria

What, then, is the source of the acyl groups in APQ or PQB, which are esterified mainly with 16:0 and/or 18:0 (**Figures 3**, **4**)? In cyanobacteria, fatty acids are synthesized as 16:0- and 18:0-acyl carrier protein (ACP) by type-2 fatty acid synthase (FASII) (Mills et al., 2020). Slr2103 or its orthologs would likely transfer newly synthesized 16:0 and/or 18:0, though it remains unclear what type of acyl donor substrate they utilize - whether it is acyl-ACP or another type of acyl-acyl carrier. In cyanobacteria such as *Synechocystis*, 18:0 and 16:0 are predominantly assembled into phosphatidate at the *sn*-1 and *sn*-2 positions, respectively, which is subsequently converted into major membrane glycerolipids (Sato and Wada, 2009). It is regarded that 18:0-ACP is subjected to conversion into 18:0-phosphate by phosphate acyltransferase (PlsX), which is then used in the reaction catalyzed by glycerol-3-phosphate acyltransferase to synthesize lysophosphatidate with 18:0 at the *sn*-1 position (Mills et al., 2020). In contrast, 16:0-ACP bypasses the PlsX reaction and is directly used in the lysophosphatidate acyltransferase [PlsC; (Okazaki et al., 2006)] reaction to synthesize PA with 18:0 and 16:0 at the *sn*-1 and *sn*-2 positions, respectively. The near-exclusive action of phosphate acyltransferase on 18:0-ACP but not on 16:0-ACP would rule out the possibility that Slr2103 or its orthologs utilize acyl-phosphate as an acyl donor substrate.

Initially, Slr2103 was found as a homolog of DGAT2, which uses acyl-CoA as an acyl donor substrate (Liu et al., 2012). *Synechocystis* possesses Slr1609 that functions as acyl-ACP synthetase, despite its homology to known acyl-CoA synthetases (Kaczmarzyk and Fulda, 2010). Moreover, *Synechocystis* lacks genes for β-oxidation, a process in which acyl-CoA is sequentially degraded to generate reducing power for ATP synthesis (Summary of Synechocystis sp. PCC 6803 substr. Kazusa, version 28.5 Tier 2 Curated Database). This suggests a relatively low metabolic demand for acyl-CoA as an acyl carrier substrate compared to β-oxidation-performing bacteria. Future studies should aim to determine whether the acyl donor substrate for Slr2103 and its orthologs is acyl-ACP or acyl-CoA. If acyl-CoA is used, it will be essential to identify novel acyl-CoA synthetase genes in cyanobacteria.

### APQ and PQB synthesis - acyl-donor synthesis in eukaryotic photosynthetic organisms

Red and green algae, and seed plants, like cyanobacteria, employ FASII in plastids to synthesize major part of cellular fatty acids (Mori et al., 2016; Hölzl and Dörmann, 2019; Li-Beisson et al., 2019). Meanwhile, *P. haptonemofera*, like its closely related species *Emiliania huxleyi*, would carry plastid-localized FASII genes (Genome assembly Emiliana huxleyi CCMP1516 main genome assembly v1.0). It is thus likely that plastid FASII, similar to cyanobacterial one, directly or indirectly provides *de novo* saturated fatty acids for APQ and PQB synthesis in these eukaryotic photosynthetic organisms. Interestingly, in *C. merolae*, PQB contained a relatively high proportion (17.4%) of 18:2-species. FASII-derived 18:0 is predominantly exported from plastids having no desaturases to the desaturase-equipped ER, where it undergoes desaturation to 18:2 (Mori et al., 2016). The resulting 18:2 is then transported to plastids, and is used for the synthesis of plastid glycerolipids. It is therefore likely that 18:2-PQB synthesis in plastids relies on 18:2 supplied from the ER. It was also exceptional that 18:1-species constituted a relatively high proportion (11.7%) of APQ in *C. reinhardtii*. In green algae such as *C. reinhardtii* and *C. kessleri*, and seed plants, 18:0-ACP synthesized by FASII undergoes highly efficient desaturation to 18:1-ACP in plastids, and the resulting acyl group is subsequently utilized for plastid and ER glycerolipid synthesis (Sato et al., 2003; Hölzl and Dörmann, 2019; Li-Beisson et al., 2019). Under our culturing conditions, 18:1-ACP in *C. reinhardtii* might have incidentally contributed to APQ synthesis. Nevertheless, it appears likely that the synthesis of APQ and PQB is completed within plastids in the green lineage.

Information on the physiological roles of APQ and/or PQB metabolism has just begun to emerge in cyanobacteria (Kondo et al., 2023b; Kondo et al., 2023a; Jimbo et al., 2024). Kondo et al. (2023b) demonstrated that, in *Synechocystis*, the level of acylplastoquinone species increased under high-salt stress conditions in static culture, contributing to the formation of pellicle biofilms on the culture surface. Pellicle biofilms would enhance the cells’ ability to acquire CO_2_ and light energy for photosynthesis, sustaining high-salt acclimating cell growth. Meanwhile, Jimbo et al. (2024) showed that, in *Synechocystis*, APL that deacylates APQ was responsible for cell growth under high light conditions (300 μmol E·m^−2^·s^−1^) and for PSII recovery from severe photodamage induced by very high light (2500 μmol E·m^−2^·s^−1^) under no aeration conditions. Our study emphasizes the need for future research to investigate the conservation of the roles of acylplastoquinone species in acclimation to high-salt or strong-light stress across a broader taxonomic range, including eukaryotic photosynthetic organisms.

## Data Availability

The raw data supporting the conclusions of this article will be made available by the authors, without under reservation.

## References

[B1] Agarwal P. Soni R. Kaur P. Madan A. Mishra R. Pandey J.. (2022). Cyanobacteria as a promising alternative for sustainable environment: synthesis of biofuel and biodegradable plastics. Front. Microbiol. 13. doi: 10.3389/fmicb.2022.939347 PMC9325326 35903468

[B2] Aizouq M. Peisker H. Gutbrod K. Melzer M. Hölzl G. Dörmann P. (2020). Triacylglycerol and phytyl ester synthesis in *Synechocystis* sp. PCC6803. Proc. Natl. Acad. Sci. U.S.A. 117, 6216–6222. doi: 10.1073/pnas.1915930117 32123083 PMC7084069

[B3] Bligh E. G. Dyer W. J. (1959). A rapid method of total lipid extraction and purification. Can. J. Biochem. Physiol. 37, 911–017. doi: 10.1139/m62-029 13671378

[B4] Bondioli P. Della Bella L. Rivolta G. Chini Zittelli G. Bassi N. Rodolfi L.. (2012). Oil production by the marine microalgae *Nannochloropsis* sp. F&M-M24 and *Tetraselmis suecica* F&M-M33. Bioresour Technol. 114, 567–572. doi: 10.1016/j.biortech.2012.02.123 22459965

[B5] Crane F. L. (2010). Discovery of plastoquinones: a personal perspective. Photosynth Res. 103, 195–209. doi: 10.1007/s11120-010-9537-9 20217233

[B6] Das B. C. Lounasmaa M. Tendille C. Lederer (1967). The structure of the plastoquinones B and C. Biochem. Biophys. Res. Commun. 26, 211–215. doi: 10.1016/0006-291x(67)90236-7 6030266

[B7] Genome assembly *Emiliana huxleyi* CCMP1516 main genome assembly v1.0. Available online at: https://www.ncbi.nlm.nih.gov/datasets/genome/GCF_000372725.1/ (Accessed 30 January).

[B8] Guillard R. R. L. Ryther J. H. (1962). Studies of marine planktonic diatoms. I. *Cyclotella nana* Hustedt, and *Detonula confervacea* (Cleve) Gran. Can. J. Microbiol. 8, 229–239. doi: 10.1139/m62-029 13902807

[B9] Havaux M. (2020). Plastoquinone in and beyond photosynthesis. Trends Plant Sci. 25, 1252–1265. doi: 10.1016/j.tplants.2020.06.011 32713776

[B10] Hayashi T. Otaki R. Hirai K. Tsuzuki M. Sato N. (2017). Optimization of seawater-based triacylglycerol accumulation in a freshwater green alga, *Chlorella kessleri*, through simultaneous imposition of lowered-temperature and enhanced-light intensity. Algal Res. 28, 100–107. doi: 10.1016/j.algal.2017.10.016

[B11] Hirabaru C. Izumo A. Fujiwara S. Tadokoro Y. Shimonaga T. Konishi M.. (2010). The primitive rhodophyte *Cyanidioschyzon merolae* contains a semiamylopectin-type, but not an amylose-type, alpha-glucan. Plant Cell Physiol. 51, 682–693. doi: 10.1093/pcp/pcq046 20385610

[B12] Holm-Hansen O. Gerloff G. C. Skoog F. (1954). Cobalt as an essential element for blue-green algae. Physiol. Planta 7, 665–675. doi: 10.1111/j.1399-3054.1954.tb07727.x

[B13] Hölzl G. Dörmann P. (2019). Chloroplast lipids and their biosynthesis. Annu. Rev. Plant Biol. 70, 51–81. doi: 10.1146/annurev-arplant-050718-100202 30786236

[B14] Ishikawa T. Takano S. Tanikawa R. Fujihara T. Atsuzawa K. Kaneko Y.. (2023). Acylated plastoquinone is a novel neutral lipid accumulated in cyanobacteria. PNAS Nexus. 2, pgad092. doi: 10.1093/pnasnexus/pgad092 37152674 PMC10156143

[B15] Jimbo H. Torii M. Fujino Y. Tanase Y. Kurima K. Sato N.. (2024). Acyl-turnover of acylplastoquinol enhances recovery of photodamaged PSII in *Synechocystis* . Plant J. 120, 1317–1325. doi: 10.1111/tpj.17051 39388621

[B16] Kaczmarzyk D. Fulda M. (2010). Fatty acid activation in cyanobacteria mediated by acyl-acyl carrier protein synthetase enables fatty acid recycling. Plant Physiol. 152, 1598–1610. doi: 10.1104/pp.109.148007 20061450 PMC2832271

[B17] Kondo M. Aoki M. Hirai K. Ito R. Tsuzuki M. Sato N. (2023a). Plastoquinone lipids: their synthesis via a bifunctional gene and physiological function in a euryhaline cyanobacterium, *Synechococcus* sp. PCC 7002. Microorganisms. 11, 1177. doi: 10.3390/microorganisms11051177 37317151 PMC10223999

[B18] Kondo M. Aoki M. Hirai K. Sagami T. Ito R. Tsuzuki M.. (2023b). *slr2103*, a homolog of type-2 diacylglycerol acyltransferase genes, for plastoquinone-related neutral lipid synthesis and NaCl-stress acclimatization in a cyanobacterium, *Synechocystis* sp. PCC 6803. Front. Plant Sci. 14. doi: 10.3389/fpls.2023.1181180 PMC10171310 37180399

[B19] Kruk J. Burda K. Schmid G. H. Radunz A. Strzałka K. (1998). Function of plastoquinones B and C as electron acceptors in Photosystem II and fatty acid analysis of plastoquinone B. Photosynth Res. 58, 203–209. doi: 10.1023/A:1006139227593

[B20] Kruk J. Strzałka K. (1998). Identification of plastoquinone-C in spinach and maple leaves by reverse-phase high-performance liquid chromatography. Phytochemistry 49, 2267–2271. doi: 10.1016/S0031-9422(98)00350-1

[B21] Kruk J. Szymańska R. (2021). Singlet oxygen oxidation products of carotenoids, fatty acids and phenolic prenyllipids. J. Photochem. Photobiol. B. 216, 112148. doi: 10.1016/j.jphotobiol.2021.112148 33556703

[B22] Kruk J. Trebst A. (2008). Plastoquinol as a singlet oxygen scavenger in photosystem II. Biochim. Biophys. Acta 1777, 154–162. doi: 10.1016/j.bbabio.2007.10.008 18005659

[B23] Ksas B. Légeret B. Ferretti U. Chevalier A. Pospíšil P. Alric J.. (2018). The plastoquinone pool outside the thylakoid membrane serves in plant photoprotection as a reservoir of singlet oxygen scavengers. Plant Cell Environ. 41, 2277–2287. doi: 10.1111/pce.13202 29601642

[B24] Li-Beisson Y. Thelen J. J. Fedosejevs E. Harwood J. L. (2019). The lipid biochemistry of eukaryotic algae. Prog. Lipid Res. 74, 31–68. doi: 10.1016/j.plipres.2019.01.003 30703388

[B25] Liu Q. Siloto R. M. Lehner R. Stone S. J. Weselake R. J. (2012). Acyl-CoA:diacylglycerol acyltransferase: molecular biology, biochemistry and biotechnology. Prog. Lipid Res. 51, 350–377. doi: 10.1016/j.plipres.2012.06.001 22705711

[B26] Mills L. A. McCormick A. J. Lea-Smith D. J. (2020). Current knowledge and recent advances in understanding metabolism of the model cyanobacterium *Synechocystis* sp. PCC 6803. Biosci. Rep. 40, BSR20193325. doi: 10.1042/BSR20193325 32149336 PMC7133116

[B27] Mori N. Moriyama T. Toyoshima M. Sato N. (2016). Construction of global acyl lipid metabolic map by comparative genomics and subcellular localization analysis in the red alga *Cyanidioschyzon merolae* . Front. Plant Sci. 7. doi: 10.3389/fpls.2016.00958 PMC4928187 27446184

[B28] Mori-Moriyama N. Yoshitomi T. Sato N. (2023). Acyl plastoquinol is a major cyanobacterial substance that co-migrates with triacylglycerol in thin-layer chromatography. Biochem. Biophys. Res. Commun. 641, 18–26. doi: 10.1016/j.bbrc.2022.12.003 36516585

[B29] Oishi Y. Otaki R. Iijima Y. Kumagai E. Aoki M. Tsuzuki M.. (2022). Diacylglyceryl-N,N,N-trimethylhomoserine-dependent lipid remodeling in a green alga, *Chlorella kessleri* . Commun. Biol. 5, 19. doi: 10.1038/s42003-021-02927-z 35017659 PMC8752610

[B30] Okazaki K. Sato N. Tsuji N. Tsuzuki M. Nishida I. (2006). The significance of C16 fatty acids in the sn-2 positions of glycerolipids in the photosynthetic growth of *Synechocystis* sp. PCC6803. Plant Physiol. 141, 546–556. doi: 10.1104/pp.105.075796 16603667 PMC1475452

[B31] Sato N. Tsuzuki M. Kawaguchi A. (2003). Glycerolipid synthesis in *Chlorella kessleri* 11h. I. Existence of a eukaryotic pathway. Biochim. Biophys. Acta 1633, 27–34. doi: 10.1016/s1388-1981(03)00069-6 12842192

[B32] Sato N. Tsuzuki M. Matsuda Y. Ehara T. Osafune T. Kawaguchi A. (1995). Isolation and characterization of mutants affected in lipid metabolism of *Chlamydomonas reinhardtii* . Eur. J. Biochem. 230, 987–993. doi: 10.1111/j.1432-1033.1995.tb20646.x 7601163

[B33] Sato N. Wada H. (2009). “Lipid biosynthesis and its regulation in cyanobacteria,” in Lipids in photosynthesis. Advances in Photosynthesis and Respiration, vol. 30 . Eds. Wada H. Murata N. (Springer, Dordrecht). doi: 10.1007/978-90-481-2863-1_8

[B34] Summary of *Synechocystis* sp. *PCC* 6803 substr. Kazusa, version 28.5 Tier 2 Curated Database. Available online at: https://cyanocyc.org/GCF_000009725/organism-summary (Accessed 30 January).

[B35] Takahashi J. Fujiwara S. Kikyo M. Hirokawa Y. Tsuzuki M. (2002). Discrimination of the cell surface of the coccolithophorid *Pleurochrysis haptonemofera* from light scattering and fluorescence after fluorescein-isothiocyanate-labeled lectin staining measured by flow cytometry. Mar. Biotechnol. (NY). 4, 94–101. doi: 10.1007/s10126-001-0083-5 14961292

[B36] Tanaka M. Ishikawa T. Tamura S. Saito Y. Kawai-Yamada M. Hihara Y. (2020). Quantitative and qualitative analyses of triacylglycerol production in the wild-type cyanobacterium *Synechocystis* sp. PCC 6803 and the strain expressing *AtfA* from *Acinetobacter baylyi* ADP1. Plant Cell Physiol. 61, 1537–1547. doi: 10.1093/pcp/pcaa069 32433767

[B37] Tanikawa R. Sakaguchi H. Ishikawa T. Hihara Y. (2025). Accumulation of acyl plastoquinol and triacylglycerol in six cyanobacterial species with different sets of genes encoding type-2 diacylglycerol acyltransferase-like proteins. Plant Cell Physiol. 66, 15–22. doi: 10.1093/pcp/pcae137 39581854

