# Supplementary material for: Evolutionary conservation of acylplastoquinone species from cyanobacteria to eukaryotic photosynthetic organisms of green and red lineages
Source: Front Plant Sci. 2025 Mar 24;16:1569038. doi: 10.3389/fpls.2025.1569038 (PMC11973298; doi:10.3389/fpls.2025.1569038)
Supplement: Supplementary file 1 [file DataSheet1.pdf]

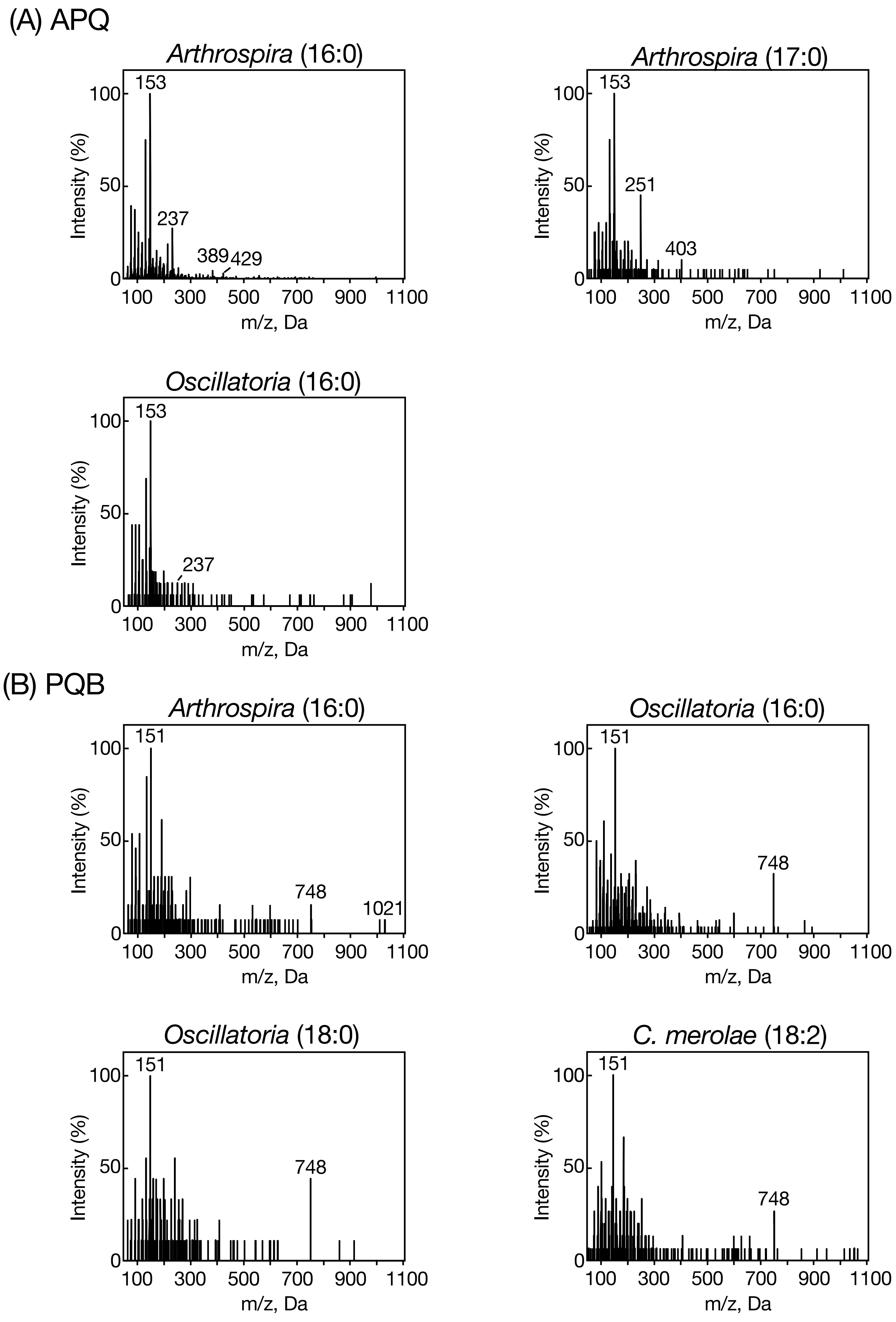

Fig. 1S MS^2 spectra of APQ and PQB molecular species in *Arthrospira*, *Oscillatoria*, and *C. merolae*.
