# Supplementary material for: Evolutionary conservation of acylplastoquinone species from cyanobacteria to eukaryotic photosynthetic organisms of green and red lineages
Source: Front Plant Sci. 2025 Mar 24;16:1569038. doi: 10.3389/fpls.2025.1569038 (PMC11973298; doi:10.3389/fpls.2025.1569038)
Supplement: Supplementary file 2 [file DataSheet2.pdf]

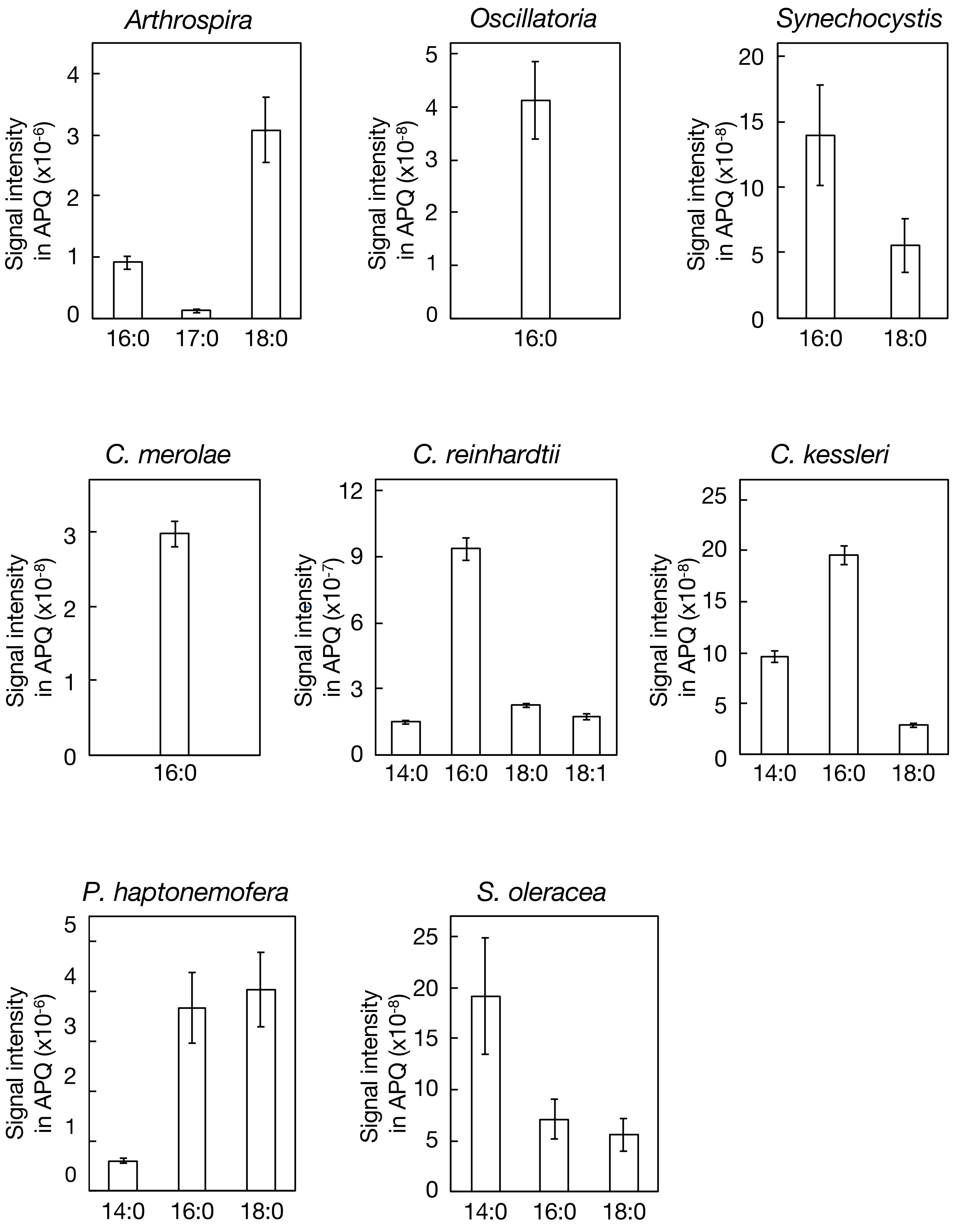

Fig. 2S Signal intensities of APQ molecular species in LC-MS spectra of total cellular lipids in respective photosynthetic organisms. The values are shown relative to the total cellular lipids, as described in Kondo et al. [7, 9].
