# Supplementary material for: Evolutionary conservation of acylplastoquinone species from cyanobacteria to eukaryotic photosynthetic organisms of green and red lineages
Source: Front Plant Sci. 2025 Mar 24;16:1569038. doi: 10.3389/fpls.2025.1569038 (PMC11973298; doi:10.3389/fpls.2025.1569038)
Supplement: Supplementary file 3 [file DataSheet3.pdf]

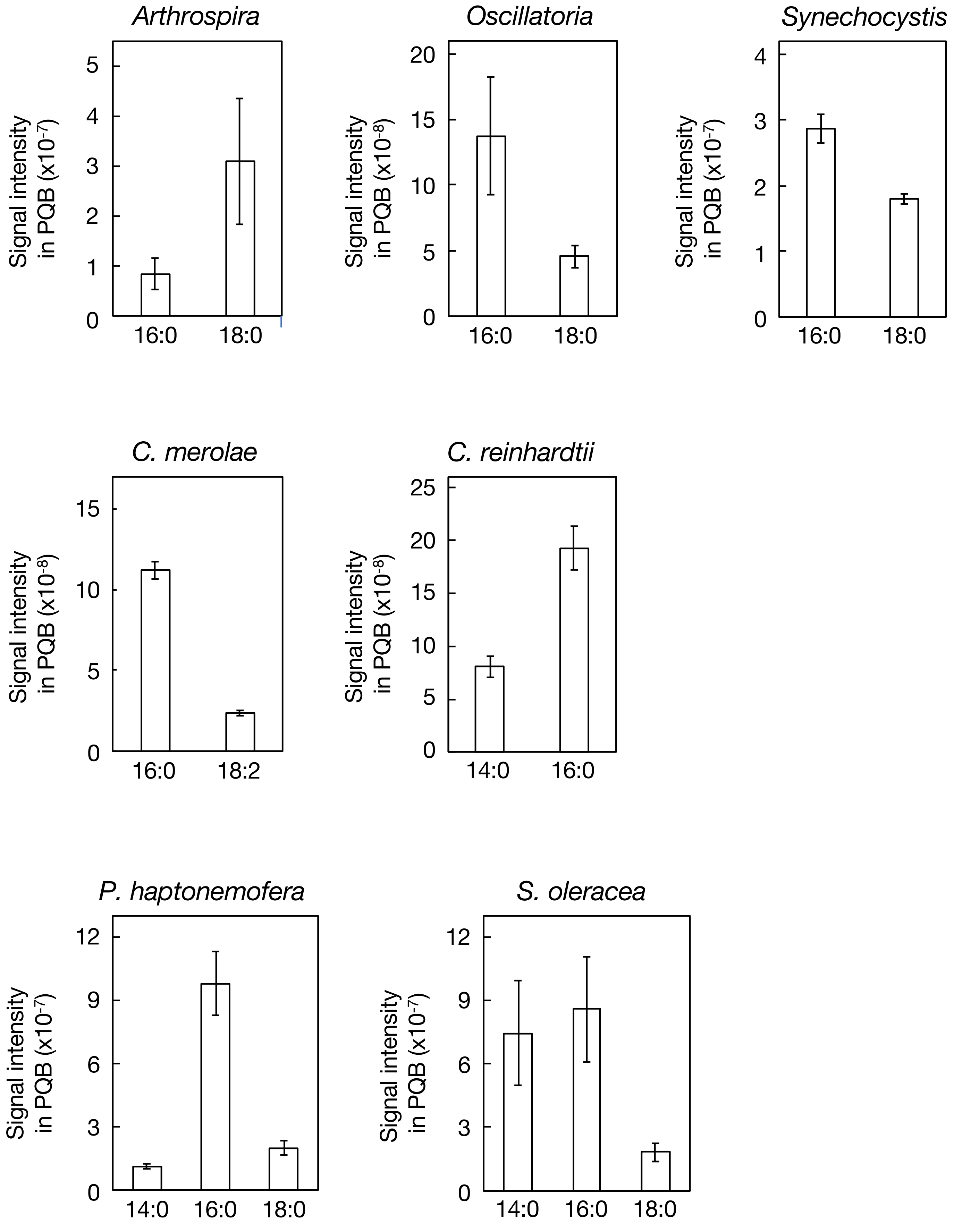

Fig. 3S Signal intensities of PQB molecular species on LC-MS spectra of total cellular lipids in the respective photosynthetic organisms. The values are shown relative to the total cellular lipids, as described in Kondo et al. [7, 9].
