# Supplementary material for: Evolutionary conservation of acylplastoquinone species from cyanobacteria to eukaryotic photosynthetic organisms of green and red lineages
Source: Front Plant Sci. 2025 Mar 24;16:1569038. doi: 10.3389/fpls.2025.1569038 (PMC11973298; doi:10.3389/fpls.2025.1569038)
Supplement: Supplementary file 4 [file DataSheet4.pdf]

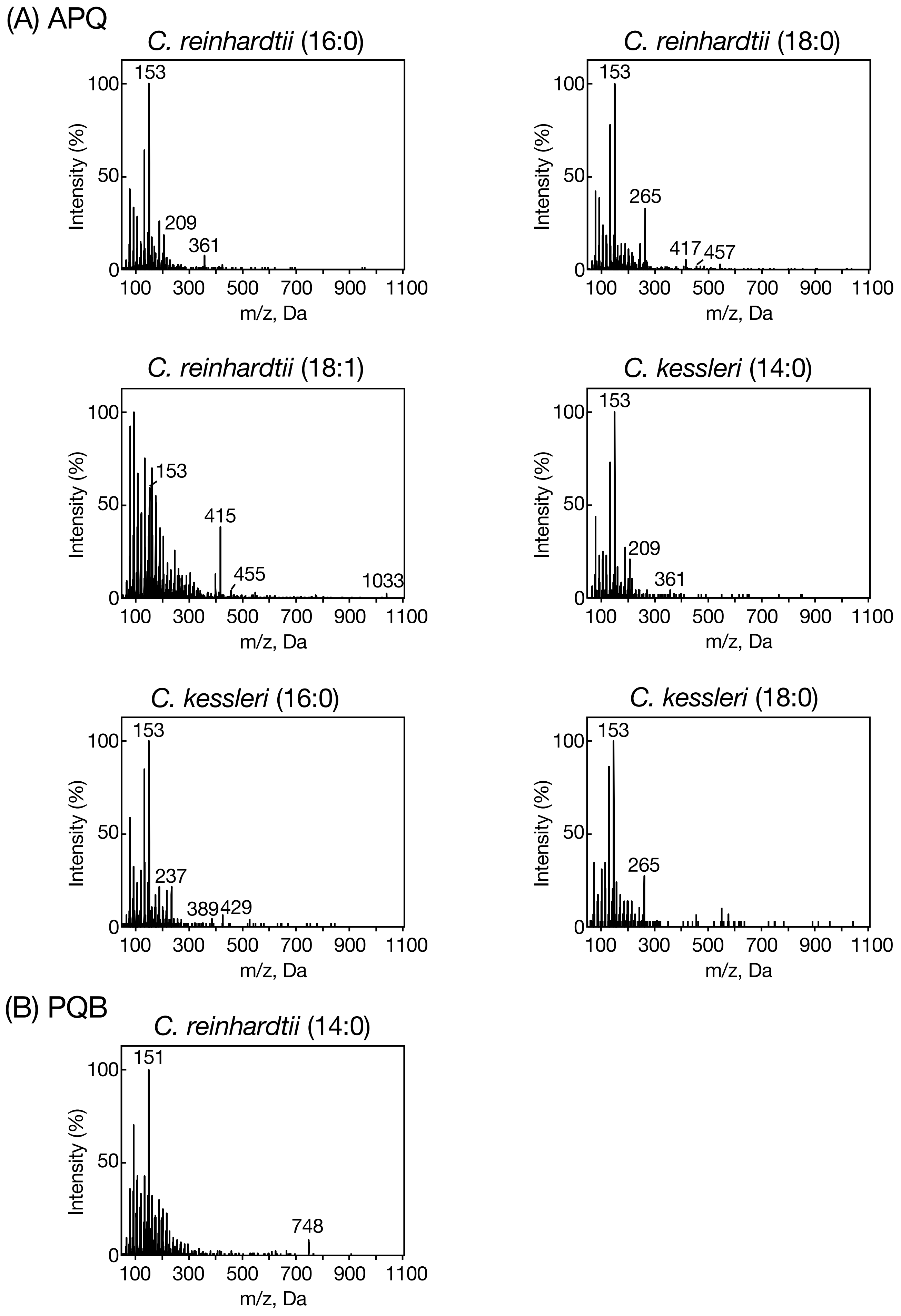

Fig. 4S MS[2] spectra of APQ and PQB molecular species in *C. reinhardtii* and *C. kessleri*.
